# International overview of phallometric testing for sexual offending behaviour and sexual risk

**DOI:** 10.1192/bji.2021.17

**Published:** 2021-11

**Authors:** Andrew Bickle, Colin Cameron, Tariq Hassan, Hira Safdar, Najat Khalifa

**Affiliations:** 1 Assistant Professor in Forensic Psychiatry, Department of Psychiatry, Queen's University, Kingston, Canada, Email: arb12@queensu.ca; 2 National Senior Psychiatrist, Correctional Service Canada/Government of Canada; 3 Associate Professor in Forensic Psychiatry, Department of Psychiatry, Queen's University, Kingston, Canada; 4 Assistant Professor, Schulich School of Medicine and Dentistry, Western University, London, Canada; 5 Associate Professor in Forensic Psychiatry, Queen's University, Department of Psychiatry, Kingston, Canada

**Keywords:** Phallometry, penile plethysmography, sex offenders, paedophilia, forensic

## Abstract

Phallometry is an objective method of assessing male sexual arousal. The main applications in forensic psychiatry concern the evaluation of men charged with or convicted of sexual offences, the evaluation of those with suspected paraphilias not subject to the criminal justice system, risk assessment and measurement of response to sex offender treatment. In some jurisdictions, phallometry is incorporated into legal decisions about release from custody or discharge from secure hospitals. This paper provides a brief overview of the international development of phallometry, considers challenges to its broader adoption and discusses future directions for research and clinical practice.

Phallometry (also known as penile plethysmography or penile tumescence testing) is an objective method of assessing male sexual arousal. In forensic psychiatry it is primarily deployed in the management of people who have committed sexual offences (PCSOs), especially those who have offended against children. Some clinics utilise phallometric testing in the management of men with suspected paraphilias not known to have acted upon their problematic sexual interests (PSIs) to commit offences. Occasionally, phallometry has been used in other settings, such family court proceedings and occupational health.

The penile plethysmograph was developed in Czechoslovakia in the 1950s by the psychiatrist Kurt Freund. At this time, when homosexuality was still illegal in many jurisdictions and regarded as a mental disorder, the technology was first directed to identifying homosexual or ‘androphilic’ desire.^1^ Although early applications included aversion therapy to ‘cure’ homosexuality, phallometric evidence of the immutability of sexual orientation supported the biological basis of homosexuality, and Freund came to lobby successfully for decriminalisation in Czechoslovakia.^2^

From the mid-1960s, phallometry was used to assess paedophilia and other PSIs. Freund emigrated to Canada in 1969 and to date phallometry has received most academic attention in that country, followed by the USA and then other countries as set out in Fig. 1. Academic interest in phallometry and offending has remained remarkably consistent over the past 30 years, with 87 publications in the 1990s, 86 in the 2000s and 88 in the 2010s.

**Fig. 1:**
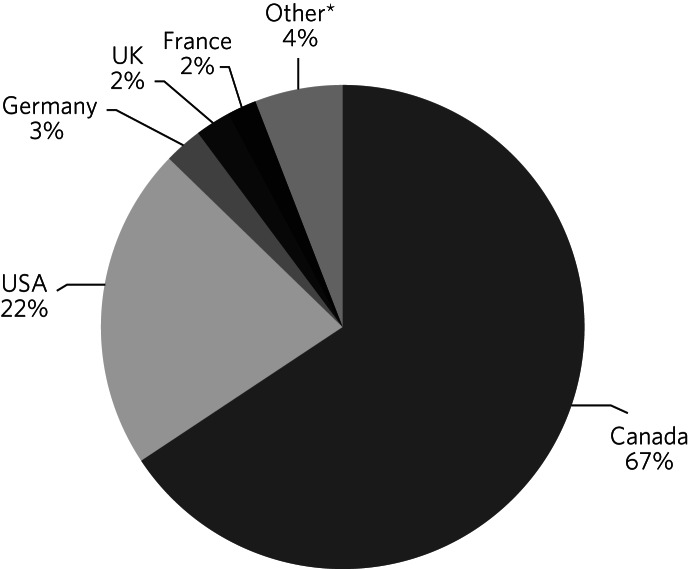
International academic interest in phallometry for offending behaviour, as measured by affiliation of first and last authors, based on a review of 291 articles identified in PubMed, PsycInfo and Embase using the terms ‘Phallometry’ OR ‘Penile Plethysmography’ OR ‘Phallometric’. *Australia 0.8%, Czech Republic/Czechoslovakia 0.8%, Netherlands 0.4%, Republic of Ireland 0.4%, Switzerland 0.4%, Belgium 0.2%, Denmark 0.2%, Portugal 0.2%, Russia, 0.2%, Singapore 0.2%, unknown 0.2%.

## Phallometry and assessment of PCSOs

When employed in clinical forensic psychiatry, phallometry should form part of a much broader sexology assessment. Phallometry response profiles should only be interpreted by experts (often forensic psychiatrists and psychologists with specific training), and the results should be used only as a supplementary resource to guide clinical impressions.^3^ Ethically, investigation should focus upon PSIs relevant to the presenting issue. Established sexual behaviour clinics, such as those in Canada and the USA, incorporate phallometry into comprehensive clinical evaluations alongside review of objective information, such as criminal records.^4^ Thornton et al^5^ recently compared phallometry for the assessment of sexual deviance with structured rating scales, indirect assessment with cognitive tasks and neuroimaging, concluding that only phallometry and scales have sufficient evidence base for their clinical use.

Phallometry may be important to the management of PCSOs because specific sexual response profiles predict recidivism^3^ and phallometric tests are valid indicators of paedophilic interests, both for PCSOs against children and controls.^6^ Thus, it is incorporated into the evaluation of PSIs, into risk assessment and into measuring response to sex offender treatment. It is mostly used to evaluate paedophilia (persistent sexual interest in prepubescent children) and to a lesser extent hebephilia (persistent sexual interest in pubescent children), although the latter has been criticised as a pathological construct lacking validity.^7^ Not all PCSOs against children have these PSIs. There may be other determinants of offending, such as easy availability of victims. Offenders without ‘paedohebephilia’ are less likely to recidivate for sexual offences against children and may have different criminogenic needs. Conversely, many people with paedohebephilic sexual interests will not act on them and commit offences.

Some non-offenders will nevertheless meet a diagnosis of ‘pedophilic disorder’ within the DSM-5^8^ owing to the marked distress or interpersonal difficulty it causes them. Phallometry may be useful in the assessment and management of this population, so it is paramount that positive findings are not equated with criminality. Other non-offender subjects may have paedohebephilic arousal without additional features warranting a diagnosis of a paraphilic disorder.

In addition, phallometry is sometimes used to assess for sexual sadism (persistent and intense sexual arousal from causing or fantasising about the physical or mental suffering of another person, with or without their consent). However, its clinical utility in this area of practice is controversial for two reasons. First, although the key features of sexual sadism are included in the current editions of the DSM^8^ and the ICD,^9^ some commentators regard sexual sadism as ‘an elusive concept to define and measure’.^10^ Second, the evidence base concerning the utility of phallometry for the identification of sexual sadism is limited, especially when considered next to the evidence concerning the identification of paedophilia. It might be surmised that the minority of violent sexual offenders who have sexual sadism are at somewhat at higher risk for recidivism, but other risk factors for violence are greater predictors of recidivism.^11^

Assessments that include phallometry are also potentially relevant to legal questions, although variation exists in adducing such evidence, even where phallometry is more commonly performed. For example, phallometric evidence is submitted to criminal sentencing in Canada more commonly than in the USA or the UK.^3^ However, in none of these countries is phallometric evidence used to determine guilt. Phallometry is also incorporated into quasi-judicial decisions about release from custody or discharge from secure hospitals.^3^

A minority of sexual offences (e.g. 5% in North America) are committed by women, and some research has objectively measured sexual arousal in female PCSOs, although this work is relatively underdeveloped.^12^ Vaginal photoplethysmography (VPP) is the most established measure of genital sexual arousal in women. Similar to phallometry, VPP measures changes in blood flow to the genitals. It utilises a photoelectric transducer within a probe akin to a menstrual tampon that is inserted into the vagina. However, the utility of VPP in identifying deviant sexual preferences in clinical forensic practice is limited by relative lack of concordance between genital and subjective sexual responses in females.^13^

## Procedure

Phallometry is conducted in specialist laboratories. There are two principal technologies: volumetric assessment and circumferential assessment. Volumetric assessment entails fitting a sealed tube device over the penis so that changes in length and diameter are detected as air is displaced from the tube. This technique is more sensitive to changes in tumescence but has several disadvantages, including the requirement for greater technician training and for the device to be technician-fitted. In circumferential assessments, a circular gauge of flexible metal or rubber, which can be fitted over the shaft by the examinee, measures change in penile girth. These are much more commonly utilised than volumetric assessments.^14^

The examinee's attention is directed to stimuli that typically include non-sexual stimuli, sexual scenarios depicting consenting adults and sexual scenarios depicting the PSI under consideration. Some facilities augment their protocols with ratings by examinees of their level of sexual arousal to each scenario. This helps to determine the reliability of subjective reporting of sexual arousal/attraction. Stimulus sets commonly include slides, videos, auditory commentaries or a combination of these. Allowing for detumescence between stimuli, testing can take several hours to complete.^4^

Results from circumferential assessment are measured in millimetres of change. A minimum significant change is applied, and scores are transformed into either percentage of full erection or standardised changes in circumference using z-scores. This reduces variability and allows within-subject and between-subjects comparisons.^14^ Scores are sometimes converted to indices of the intensity of paraphilic interest relative to normophilic sexual interests (e.g. sexual interest in consenting adults).

In the modern history of phallometry, research adaptations have included testing the examinee when he is intoxicated to simulate mental state at the time of the offence,^15^ testing after the administration of sildenafil to promote erectile response,^16^ testing with virtual reality stimuli^17^ and simultaneous phallometry with eye-tracking.^18^ These interesting ideas have not made their way into standard clinical practice.

## Disadvantages, challenges and controversies

Limitations to phallometry might be divided into technical factors and societal factors, including ethical factors. First, as indicated, the adequate performance of testing is relatively resource intensive. Second, concerns about examinee manipulation of phallometric testing (either by suppressing socially undesirable responses or simulating responses to normophilic stimuli) are longstanding. Successful manipulation plainly reduces the sensitivity of the test. However, Babchishin et al^19^ found from a large sample that over 80% of PCSOs against children were unable to successfully inhibit their sexual arousal to children when instructed to do so. Some laboratories check examinees’ attention to the stimuli by asking them to describe it^20^ or by videoing their upper body.^14^ Third, the specificity of phallometry is an important consideration owing to the potential implications of incorrectly identifying an examinee with a PSI. Although its specificity in paedophilia has been found to be impressive (e.g. volumetric phallometry has been shown by Blanchard et al^21^ to have specificity of 96% in distinguishing men with a primary sexual interest in adults from those with such an interest in children), it is less specific for hebephilia.^22^ This stresses the importance of restricting phallometry in clinical forensic practice to one component of a comprehensive assessment for proportionately serious assessment needs. It should also heighten caution in assessing sexual interest in pubescent children which, unlike sexual interest in prepubescent children, is not recognised in the DSM-5 as forming the basis of a paraphilic disorder.

Other technical concerns have included the potential for medications to interfere with penile response. However, Lykins et al^23^ found that differences in those taking psychotropic and other medications were largely age-related.

Regarding societal factors, the intrusive nature of phallometry raises concerns, although the circumferential gauge method allows the examinee to fit the device himself. Such concerns resulted in phallometry being banned in UK prisons in the 1980s, for example, although this was reversed a few years later when evidence-based treatment programmes for sex offenders were introduced.^3^

Moreover, there are ethical concerns about examinees being coerced into testing when a court orders it. Proponents argue that only the meeting with the psychiatrists is mandated and phallometry is done with informed consent (e.g. Ref. 14). However, Purcell et al^24^ highlight that a court might draw adverse inference from the unwillingness of the defendant to participate in phallometric testing alone. This may be mitigated somewhat if testing is deferred until after a verdict has been rendered.

Other challenges arise from lack of standardisation in testing procedures, including choice of assessment stimuli, assessment devices, measurement cut-offs, testing rooms, and assessment protocols, as well as analysis and interpretation of results (for comprehensive reviews, see Refs 14,25). The choice of stimuli presented in phallometric testing, for instance, varies across jurisdictions. In Canada, use of nude images of all age groups is permitted for clinical assessment and research purposes.^14^ By contrast, in many countries, such as the USA and the UK, prohibition of nude images of children extends to clinical and research use, giving some indication of the ethical hazards in this space and concerns about victimisation of child models. Adaptations might include pictures of children in swimsuits, computer-generated images or greater reliance upon auditory stimuli, which in turn can be synthesised. Restrictions in other countries may be more significant still. Indeed, Carvalho et al^20^ speculate that legal regulations concerning use of stimuli may explain why the majority of research on phallometry is from North America and parts of continental Europe.

Another limitation regarding the generalisability of current research is that it has mainly involved White men,^7^ so more transcultural studies are needed.

## Future directions

Are circumstances propitious for this established test to be utilised worldwide? Further academic work might facilitate more extensive adoption. The development in isolation of different assessment protocols (including types of equipment, stimulus sets, analysis and interpretation) at individual laboratories has challenged replication and robust comparison. There is now reported an international effort by experts from Canada, the USA, the UK, the Czech Republic and Russia to standardise the assessment and treatment of PSIs and behaviours,^26^ offering the prospect of a standardised protocol for phallometry laboratories. There would remain variation in the permissibility of test stimuli in different jurisdictions. However, it seems conceivable that an evidence-based hierarchy of stimulus sets could be set down, at least.

Elsewhere, there appears to be an absence of research into the acceptability of phallometry, including qualitative research on the attitudes of examinees or professionals to phallometry. Such work might identify barriers to usage that could be addressed and better articulate ethical concerns.

In summary, phallometry might be regarded as an unusual aspect of psychiatric practice, and its relatively modest adoption internationally despite decades of use in some countries is therefore unsurprising. However, contemporary work to make more robust both the evidential basis and practical application of the test may promote acceptance and greater deployment in years to come.

## Supplementary material

For supplementary material accompanying this paper visit https://doi.org/10.1192/bji.2021.17. click here to view supplementary material
